# Mechanical Behaviors and Fatigue Performances of Ballastless Tracks Laid on Long-Span Cable-Stayed Bridges with Different Arrangements

**DOI:** 10.3390/s19194195

**Published:** 2019-09-27

**Authors:** Xingwang Sheng, Weiqi Zheng, Zhihui Zhu

**Affiliations:** 1 School of Civil Engineering, Central South University, Changsha 410075, China; shengxingwang@163.com (X.S.); zzhh0703@163.com (Z.Z.); 2 National Engineering Laboratory for High-Speed Railway Construction, Changsha 410075, China

**Keywords:** high-speed railway, long-span cable-stayed bridge, ballastless track, full-scale fatigue test, mechanical behavior, fatigue performance, rubber isolation layer, interlayer

## Abstract

In this paper, we present a new attempt to lay ballastless tracks on long-span cable-stayed bridges on high-speed railways. The arrangements of ballastless tracks laid on cable-stayed bridges can be divided into two conditions: (i) across the cable suspension-point cross-section or (ii) in discontinuity at the cable suspension-point cross-section. At present, there is a lack of in-depth research on ballastless tracks laid on long-span cable-stayed bridges, especially on the mechanical behaviors and fatigue performances of the ballastless tracks with different arrangements. For this paper, a segmental model of a long-span cable-stayed bridge was designed and built, on which full-scale ballastless tracks with two different arrangements were arranged. A series of fatigue tests and post-fatigue loading tests were carried out based on the two selected full-scale ballastless tracks. Some conclusions were drawn as follows. For the longitudinal end of the ballastless track, which is far from the loading positions, the interlayers of the ballastless tracks tend to warp up relatively, and the compressive pressures at the interlayers are also unloaded. However, there is no void or gap formed at the interlayers of the longitudinal end of the track slab due to the precompression of the rubber isolation layer. For the center of the track slab, which is close to the loading positions, the compressive deformations occur at the interlayers, and the pressures at interlayers are also increased. The maximum compressive deformation is less than 0.5 mm under the standard train axle load (170 kN), and it cannot affect the high-speed trains’ operation. With the increase of the post-fatigue loading, the load-displacement curves and the load-pressure variation curves of the ballastless tracks show apparent nonlinearity. Moreover, with the increase of the fatigue loading cycles, the compressive stiffness enhancement or degradation of the ballastless tracks are not noticeable. That is to say, the ballastless tracks laid on the long-span cable-stayed bridges with different arrangements have good mechanical behaviors, and their fatigue performances can also be guaranteed after bearing repeated loadings.

## 1. Introduction

Due to numerous advantages, including lighter structural deadweight, lower maintenance costs, and increased service life, ballastless tracks have been widely used in the construction of roadbeds, tunnels, and common-span bridges on high-speed railways [1,2,3,4]. Currently, a series of studies have been focused on the mechanical properties [5,6,7,8,9,10] and the dynamic characteristics [11,12,13,14,15] of ballastless tracks. Many achievements have been made, and some improvements have been applied to ballastless track structures. However, the ballastless tracks have so far never been laid on long-span cable-stayed bridges on high-speed railways due to technical constraints.

According to the authors’ knowledge, the study of ballastless tracks laid on long-span bridges is very rare [16,17]. Unfortunately, for long-span bridges such as the Tianxinzhou Bridge on the Wuhan–Guangzhou high-speed railway and the Dashengguan Bridge on the Beijing–Shanghai high-speed railway, ballasted track is usually laid on these high-speed railway long-span bridges, but the ballastless track is always laid on the approach bridges on both sides of these long-span bridges. As a result, this results in excessive transitions between the ballasted tracks and the ballastless tracks, which is inconvenient for the operation and maintenance of the track structures. Evidently, it is of considerable significance to lay ballastless tracks on the high-speed railway long-span bridges and their approach bridges.

Moreover, long-span cable-stayed bridges have many suspension points, which lead to a series of complex deformation conditions of the cable-stayed bridges. Obviously, the deformation and stress characteristics of the ballastless tracks laid on the long-span cable-stayed bridges are significantly different from those ballastless tracks laid on roadbeds, tunnels, and common-span bridges [18]. Typically, the arrangement of the ballastless tracks laid on cable-stayed bridges can be divided into two conditions: (i) across the cable suspension-point cross-section or (ii) in discontinuity at the cable suspension-point cross-section. Therefore, the mechanical behaviors and fatigue performances of ballastless tracks with two different arrangements laid on long-span cable-stayed bridges must be studied in depth.

In situ tests and segmental model tests were considered as the practical approaches to research the mechanical characteristics of the present large-scale structures [19,20,21,22,23,24,25]. In this paper, a segmental model of a long-span cable-stayed high-speed railway bridge was designed and built in the laboratory, and then a series of full-scale fatigue tests and post-fatigue loading tests of the ballastless tracks with different arrangements laid on the segmental model were carried out. The deformation and pressure variations at the interlayers were measured during the post-fatigue static loading procedure to explore the mechanical behaviors and fatigue performances of ballastless tracks laid on long-span cable-stayed bridges.

## 2. Long-Span Cable-Stayed Bridge and Ballastless Tracks

**Figure 1:**
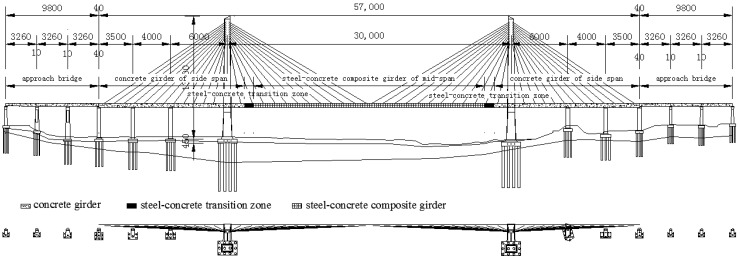
Ganjiang Bridge (unit: cm).

At present, ballastless tracks have never been laid on long-span cable-stayed bridges on high-speed railways. Moreover, the feasibility of laying ballastless tracks on long-span cable-stayed bridges deserves to be studied further. In this paper, some studies were carried out based on a newly built long-span cable-stayed bridge called the Ganjiang Bridge on the Nanchang–Ganzhou high-speed railway in China. The Ganjiang Bridge is a long-span steel-concrete composite-girder cable-stayed bridge with the span arrangement of 35 + 40 + 60 + 300 + 60 + 40 + 35 m (Figure 1). The middle span of the Ganjiang Bridge is the steel-concrete composite box girder, and the side span is the prestressed concrete box girder. The cables on the bridge are evenly arranged, and the spacing of the two adjacent cables is 12 m. The arrangement of the ballastless tracks on the Ganjiang Bridge has not yet been formally determined, and the related research is still underway. This work is focused on this research.

**Figure 2:**
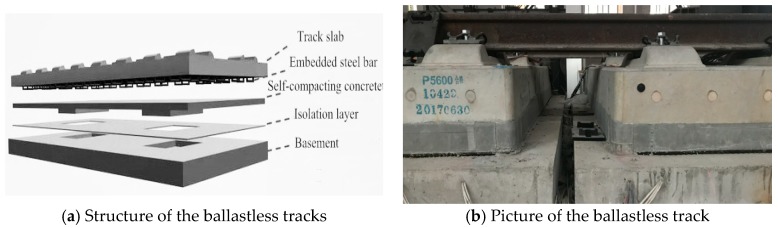
CRTS III slab ballastless track: (**a**) structure, (**b**) picture.

The Ganjiang Bridge is designed to allow the laying of CRTS III slab ballastless tracks, becoming the first long-span cable-stayed high-speed railway bridge with ballastless tracks in the world. The CRTS III slab ballastless tracks are discontinuous, and consist [26,27] of rails, a fastener system, a concrete track slab, a self-compacting concrete filling layer, an isolation layer, and a concrete basement from top to bottom (Figure 2). 

**Figure 3:**
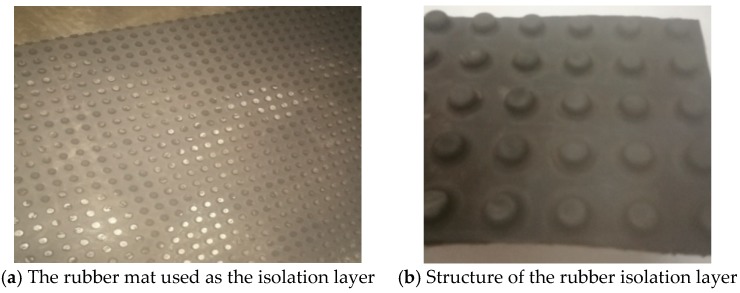
Rubber isolation layer: (**a**) rubber mat, (**b**) structure.

In the CRTS III slab ballastless track structure, the isolation layer injected between the concrete basement and the track slab is one of the most important parts. The isolation layer has the function of deformation coordination and vibration reduction due to its low compressive stiffness and large elasticity [28,29,30]. The adequate arrangement of the isolation layer can avoid voids and gaps formed at the interlayers of the ballastless tracks. What requires special attention is the ethylene propylene diene monomer (EPDM) mat [31,32,33,34] that was used as the isolation layer of the CRTS III slab ballastless track in this research (Figure 3). Some research has been done on the EPDM mat’s specifications for CRTS III slab ballastless track applications [32]. In this work, the thickness of the EPDM isolation layer (or rubber isolation layer) was 14 mm, and the stiffness was 0.1 N/mm^3^. The rubber isolation layer was laid on the basement of the ballastless track, and then the self-compacting concrete filling layer was poured directly on the rubber isolation layer.

## 3. Test Preparation

### 3.1. Segmental Model and Ballastless Tracks

**Figure 4:**
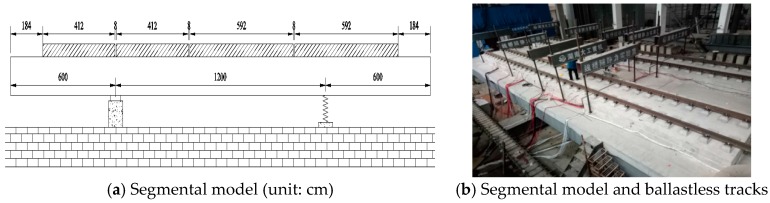
Segmental model of the Ganjiang Bridge: (**a**) model, (**b**) model and tracks.

In this paper, the equivalent segmental model of the Ganjiang Bridge was built in the laboratory in order to study the mechanical behaviors and fatigue performances of ballastless tracks laid on long-span cable-stayed bridges with different arrangements. The segmental model was simplified and equivalently designed based on the mid-span girder of the Ganjiang Bridge. The total length of the segmental model was 24 m, the width was 10 m, and the height was 1.5 m. There were two supporting cross-sections under the segmental model, one of which was rigid support (i.e., concrete pier) and the other was elastic support (i.e., elastic steel cross-beam under the beam to simulate the elastic fulcrum of the Ganjiang Bridge) (Figure 4).

**Figure 5:**
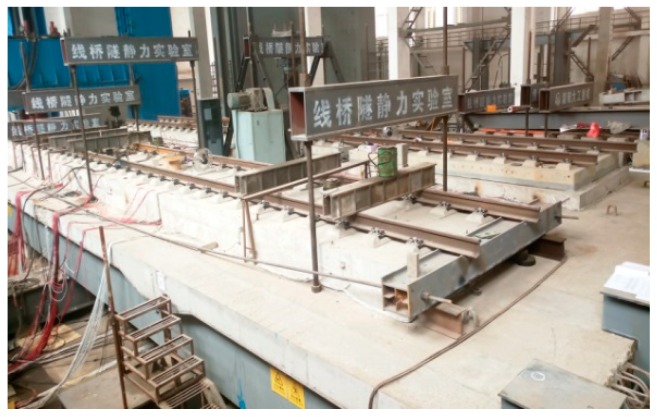
Full-scale ballastless tracks.

**Figure 6:**
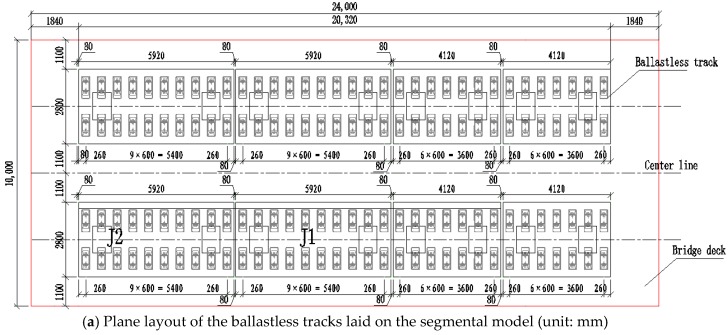

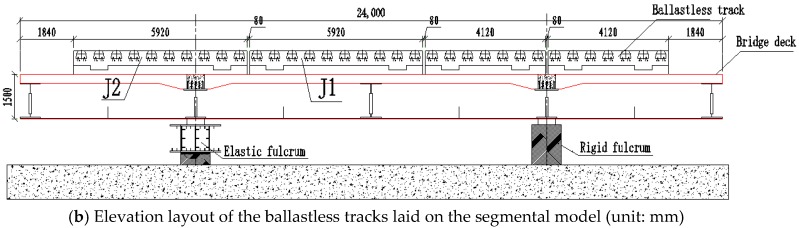
The (**a**) plane and (**b**) elevation layouts of the ballastless tracks laid on the segmental model.

Eight blocks of ballastless tracks were arranged on the segmental model, divided into two lines (Figure 5). Two blocks of the CRTS III slab ballastless tracks were selected as the research objects in this work. One of the selected ballastless tracks was arranged between the two adjacent cable suspension points (the two supporting sections of the segmental model), labeled as J1. The other was arranged across the section of the cable suspension point (the elastic supporting section of the segmental model), labeled as J2. The two CRTS III slab ballastless tracks used for full-scale fatigue tests and post-fatigue loading tests are shown in Figure 6.

The material of the steel box girder of the segmental model was Q345, and the material of the prefabricated concrete bridge deck of the segmental model was C60. The steel-concrete composite girder was formed by connecting the steel box girder and the prefabricated concrete bridge deck through cast-in-place wet joints. The material of the cast-in-place wet joints was C55 shrinkage compensating concrete. For the CRTS III slab ballastless track, its track slab was C60 concrete with a thickness of 26 cm, and the basement was C40 concrete with a thickness of 24 cm. The steel bars used in the ballastless track and prefabricated concrete bridge deck were HRB500 steel bars. The strength grade of the self-compacting concrete filing layer in the CRTS III slab ballastless track was C40, which was specially designed to meet the filing requirements [35,36].

### 3.2. Measuring Sensors

**Figure 7:**
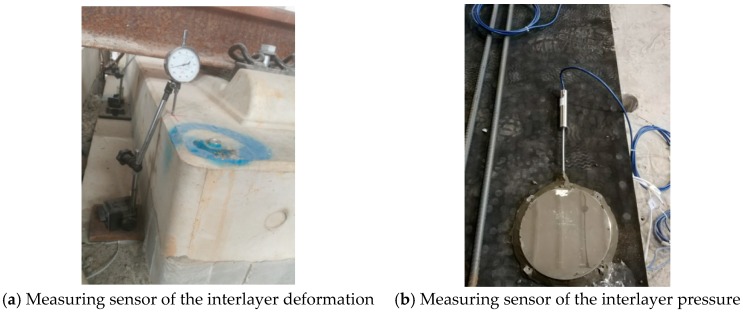
The layout of the (**a**) interlayer deformation and (**b**) interlayer pressure measuring sensors.

The variation in interlayer behaviors can reflect the mechanical characteristics of ballastless tracks laid on long-span cable-stayed bridges with different arrangements. That is to say, researchers should focus on the change in mechanical properties of the ballastless tracks during the fatigue loading process [37,38]. The mechanical properties at the interlayers of ballastless tracks are difficult to obtain, and the reliability of common sensor test results is also difficult to guarantee after experiencing certain fatigue load cycles. In this work, a series of dial gauges and high-quality pressure measuring sensors were used to research the interlayer behaviors of the ballastless tracks laid on the segmental model. In detail, a series of dial gauges were placed vertically at the isolation layers of the ballastless tracks to measure the vertical displacements between the track slab and the basement during the post-fatigue loading process. Moreover, a set of high-quality pressure measuring sensors were arranged on the upper surface of the isolation layer to measure the pressure variations at the interlayers of the ballastless tracks during the fatigue tests. The layouts of the measuring sensors are shown in Figure 7.

**Figure 8:**
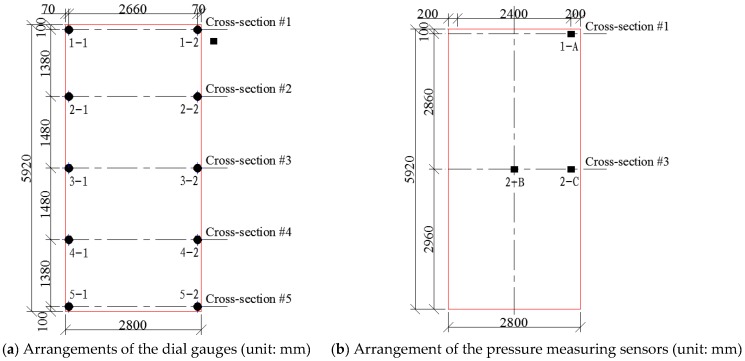
The arrangements of the (**a**) dial gauges and (**b**) pressure measuring sensors.

Ten dial gauges and three pressure measuring sensors were installed on each of the two full-scale ballastless tracks. The ten dial gauges were arranged into five cross-sections on each of the ballastless tracks, and the three pressure measuring sensors were arranged into two cross-sections. For the two ballastless tracks used in this work, the arrangements of the measuring sensors are the same. Specifically, the arrangements of the dial gauges and the pressure measuring sensors are shown in Figure 8.

The dial gauges were installed before every post-fatigue static loading test of the ballastless tracks, and the reliability of the test results can be easily guaranteed. The interlayer pressure measuring sensors were embedded in the ballastless track manufacturing process, and they should be protected with special attention. Unfortunately, for both the ballastless tracks labeled as J1 and J2, the test results of the pressure measuring sensor called 2-B were distorted, and the test results of this sensor are unreliable. The other pressure measuring sensors worked well in the whole fatigue test process, and further analysis can be carried out later.

### 3.3. Test Conditions

The finite element models of the Ganjiang Bridge and the segmental model were built based on ANSYS software (version number 14.5, ANSYS, Canonsburg, PA, USA). The deformation characteristics of the Ganjiang Bridge under various load conditions were obtained by finite element analysis, which provided the basis for the design of the fatigue tests in this research. The maximum deformation of the segmental model under the maximum fatigue loading is equal to the deformation of the main bridge within the two adjacent cables under the unfavorable combination of ZK live load and temperature effect.

**Figure 9:**
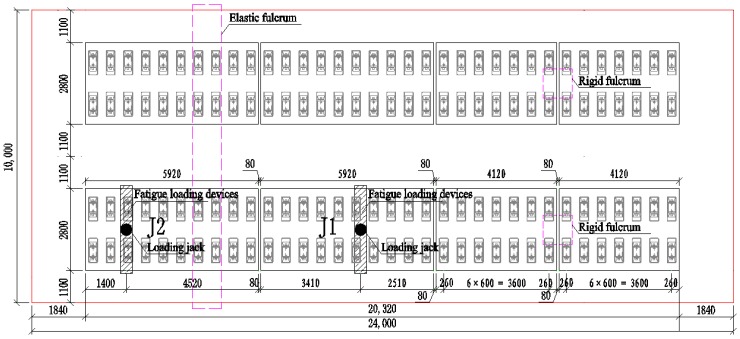
Loading positions of the two full-scale fatigue tests (unit: mm).

The two ballastless tracks used for the full-scale tests in this paper were located at different positions on the segmental model to simulate the different arrangements of ballastless tracks laid on long-span cable-stayed bridges. For the ballastless track labeled as J1, the distances from the loading position to the two longitudinal ends of the ballastless track were 2.51 m and 3.41 m, respectively. Under this loading mode, the deformation of the middle span of the segmental model under the maximum fatigue load was equivalent to that of the main beam of the Ganjiang Bridge under the most unfavorable combination of temperature and live load. For the ballastless track labeled as J2, the distance between the loading position and the two longitudinal ends of the ballastless track was 1.4 m and 4.52 m, respectively. The determination of the loading position was the same as that of the ballastless track labeled as J1. The loading positions of the two ballastless tracks used for testing are shown in Figure 9.

According to the analysis results of the finite element models, the distances of each cross-section from the loading position are summarized in Table 1. The loading locations are directly related to the mechanical behaviors of the ballastless tracks, and Table 1 is helpful in understanding the experimental results presented in this paper.

## 4. Experimental Procedure

### 4.1. Loading Devices

**Figure 10:**
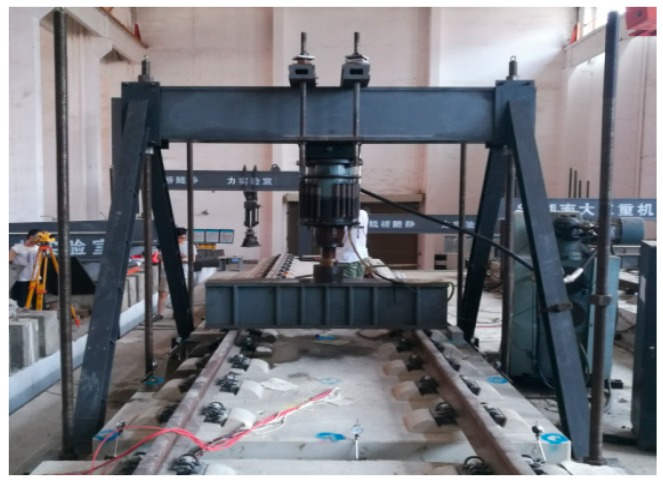
Loading device.

Due to the large scale of the segmental model and ballastless tracks, it was challenging to obtain the fatigue loading using the standard counterforce frame devices. Therefore, a set of fatigue loading devices were fabricated using high-strength screws and hot-rolling H beam steel to obtain the fatigue loading on full-scale ballastless tracks in this work. First, six holes were reserved on the segmental model, and the high-strength screws were passed through these reserved holes. Then, the lower ends of these high-strength screws were anchored onto the bottom plate of the laboratory, and the upper ends of these high-strength screws were equipped with the H-shaped steel loading device. Next, the loading device and the high-strength screws constituted a system to reduce the swing of the counterforce frame devices. Finally, the fatigue loading was obtained. The loading device is shown in Figure 10.

### 4.2. Loading Scheme

**Figure 11:**
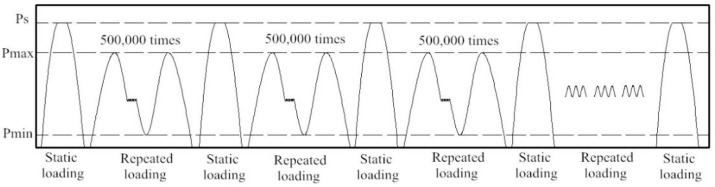
Procedure of the fatigue tests.

The upper limit load in the fatigue test was determined according to the fatigue checking load in the Code for the design of high-speed railways (TB 10621-2014) [39]. It is 1.5 times the running train axle load; in this work, it was 255 kN. The lower limit load is related to the designed secondary dead load, which was 50 kN in this work. The repeated fatigue loading cycles for the CRTS III slab ballastless tracks labeled as J1 and J2 were 5,000,000 times and 3,000,000 times, respectively. Moreover, the loading frequency of the fatigue test was between 2.4 Hz and 2.8 Hz. During the fatigue loading process, the repeated loading was first periodically paused, and the ballastless track was unloaded to zero when the fatigue cycle was ranged to 0 times (before the test), 500,000 times, 1,000,000 times, and every 500,000 times thereafter until 5,000,000 times [40]. Then the dial gauges were placed, ensuring that the pointers were in contact. Subsequently, the post-fatigue static loading tests were carried out sequentially from zero to the maximum static load Ps. That is, Ps was considered as 340 kN in this work. At the intervals of the post-fatigue static loading tests, the deformation and the pressure variation at the interlayers of the ballastless tracks were recorded when the digits of the dial gauges become steady. Moreover, a crack was observed in the web, top, and bottom slab throughout the fatigue test process. The complete loading procedure of the full-scale fatigue tests and post-fatigue loading tests are shown in Figure 11.

## 5. Test Results

### 5.1. Deformations at Interlayers

**Figure 12:**
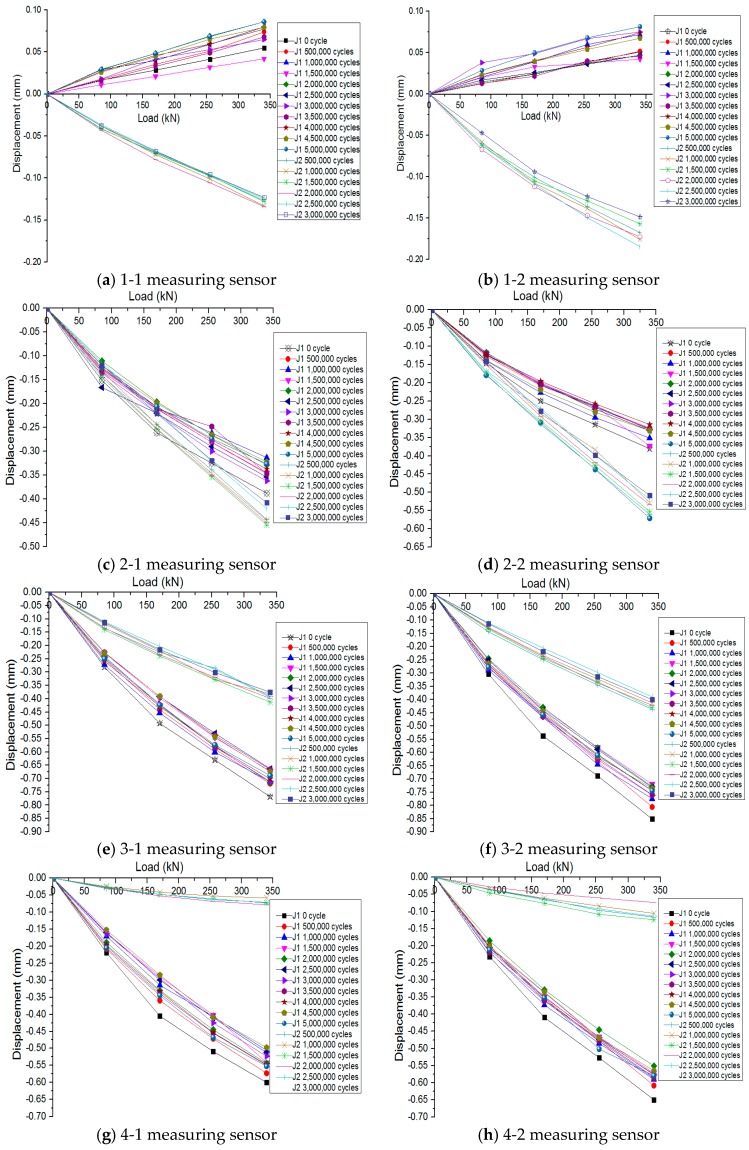

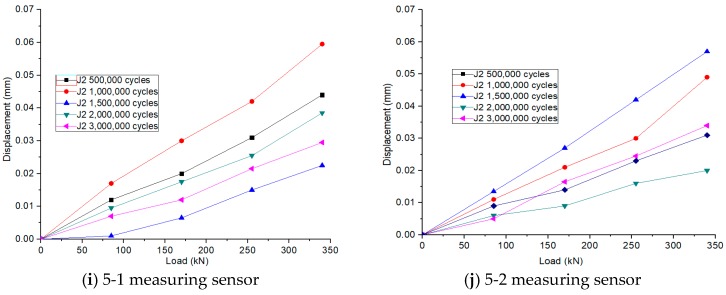
Load-displacement curves.

The deformations and pressure distributions at the interlayers of the ballastless tracks change when loads act on the rails. Interlayer deformations of the ballastless tracks with different arrangements on the segmental model were measured during the post-fatigue static loading process in this work. Moreover, the variation characteristics of the interlayer deformations under certain loading cycles were investigated. For the two full-scale ballastless tracks labeled as J1 and J2 under the post-fatigue static loading after enduring certain fatigue loading cycles, the variations in the deformations at the interlayers are shown in Figure 12, in which the interlayer compressive deformations are expressed as the negative values and interlayer tensile deformations are expressed as the positive values.

As can be seen from Figure 12, the experimental results of the two full-scale fatigue tests are very similar, and the further summary and analysis are as follows.

For the ballastless track labeled as J1, under the post-fatigue static loading, there is a tendency of tensile deformation formed at the interlayers of cross-section #1. In detail, under the standard axle load of a train in China (170 kN), the tensile deformation at the interlayers is less than 0.08 mm. For cross-sections #2, #3, and #4, some compressive deformations occur at the interlayers of the ballastless track, and the maximum compressive deformation occurs at the interlayer of cross-section #3, which is nearest to the loading position. The maximum compressive deformation is less than 0.5 mm, and it cannot affect the smoothness and safety of the operation of the high-speed trains.

For the ballastless track labeled as J2, under the action of the post-fatigue static loading, some compressive deformations occur at the interlayers of the ballastless track from cross-sections #1 to #4. The maximum compressive deformation occurs at the location of cross-section #2, which is nearest to the loading position, and the maximum compression is no more than 0.35 mm under the standard axle load of 170 kN. This value is much smaller than the limit of the rail irregularity, and it does not affect the rail alignment. Remarkably, the tensile deformation occurs at cross-section #5, which is farthest from the loading position, and the deformation value is minimal, with an average value of 0.05 mm.

With the increase of the fatigue loading cycles, the load–displacement curves of the two ballastless tracks are maintained within a range, and the difference between the load–displacement curves is not significant. It can be considered that the standard fatigue load has little effect on the ballastless tracks laid on long-span cable-stayed bridges with different arrangements. That is to say, the mechanical performances of the ballastless tracks have no obvious degradation, and the mechanical properties at the interlayer are reliable. Moreover, with the increase of the post-fatigue static loading, the load–displacement curves show a particular nonlinear characteristic. The nail structures of the rubber isolation layer are compressed and deformed under the post-fatigue static loading, which results in the larger contact area between the rubber nail structures and the concrete basement. As a result, the growth rate of the interlayer deformation becomes slower with the increase of the post-fatigue static loading. In other words, the compressive stiffness of the rubber isolation layer becomes much more significant with the static loading increases.

### 5.2. Interlayer Pressure Variations

**Figure 13:**
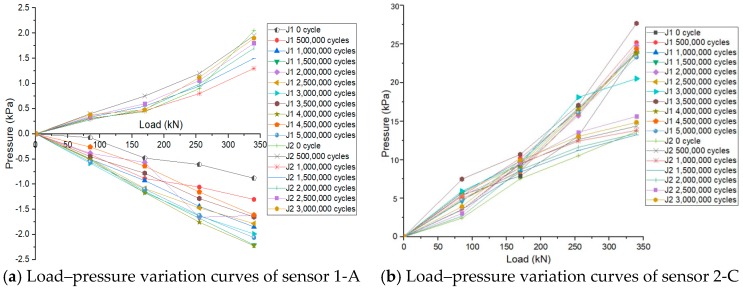
Load–pressure variation curves of measuring sensors (**a**) 1-A and (**b**) 2-C.

A small number of pressure-measuring sensors were arranged in these tests. The variations of the interlayer pressure of the two ballastless tracks labeled as J1 and J2 under the post-fatigue static load after enduring certain cycles of fatigue loading are shown in Figure 13. The increase of interlayer pressure is expressed as the positive value and the decrease of interlayer pressure is expressed as the negative value in Figure 13.

As can be seen from Figure 13, for the ballastless track labeled as J1, the interlayer pressure of sensor 1-A decreases with the increase of the post-fatigue loading, whereas the interlayer pressure of sensor 2-C increases with the increase of the post-fatigue loading. For the ballastless track labeled as J2, the interlayer pressures of the two sensors both increase with the increase of the post-fatigue loading. The interlayer pressure near the loading position increases, whereas the interlayer pressure at the positions far from the loading position tends to be decreased. The increase of pressure indicates that some compressive deformations occur at the interlayers of the ballastless tracks, and the decrease of pressure indicates that some tensile deformations occur at the interlayers of the ballastless tracks. Thus, in comparison with the test results of the interlayer deformation in Figure 12, the interlayer pressure decreases when the interlayer tensile deformation occurs, and the interlayer pressure increases when the interlayer compressive deformation occurs. The variation in the interlayer pressure is consistent with that of the interlayer deformation.

## 6. Discussion

The effects of the tensile deformations and the compressive deformations at the interlayers on the ballastless tracks are entirely different. The compressive deformations at the interlayers affect the rail alignment, and its impact on the operation of the running trains can be effectively eliminated. However, the tensile deformations at the interlayers not only affect the rail alignment but also cause some structural breakdowns at the interlayers of the ballastless tracks, such as gaps and voids. These breakdowns cause the collision between the track slab and the basement of ballastless tracks, which can accelerate the degradation of the ballastless tracks. Some reasonable measures must be taken to control the interlayer tensile deformations, so as to eliminate the interlayer breakdowns and ensure the safety and reliability of the ballastless tracks in service.

For ballastless tracks laid on long-span cable-stayed bridges with different arrangements, the compressive deformations at the interlayers decrease with the increase of the distance to the loading positions. Moreover, tensile deformations even occur at the interlayers of the longitudinal end of the ballastless tracks, which are far from the loading positions. An elastic rubber mat was arranged between the track slab and the basement of the ballastless track, and reasonable control of the elasticity of the rubber mat can solve the problem. In this work, due to the elastic rubber isolation layer laid between the self-compacting concrete filling layer and the concrete basement, the rubber isolation layer was compressed under the deadweight of its upper structures during the construction process. According to the compressive stiffness of the rubber isolation layer used in this work, the precompression of the rubber isolation layer is about 0.1 mm under the deadweight of its upper structures including track slab, self-compacting concrete filling layer, and rails. In the two full-scale fatigue tests, the maximum tensile deformation at the interlayers under the standard train axle load of 170 kN did not reach the precompression of the rubber isolation layer under the deadweight of its upper structures. The interlayers of the ballastless tracks with different arrangements can be filled by the rubber isolation layers. As a result, those breakdowns such as gaps and voids cannot be formed at the interlayers of ballastless tracks laid on long-span cable-stayed bridges with different arrangements.

Simultaneously, some strict requirements should be placed on the rubber isolation layer’s fatigue behaviors to ensure that it is safe and reliable during service. In this work, with the increase of the fatigue loading cycles, both the loading–deformation curves and the loading–pressure variation curves of the two fatigue tests were maintained within a range, and the variations were not apparent. Therefore, it can be concluded that the mechanical behaviors and fatigue performances of ballastless tracks laid on long-span cable-stayed bridges with different arrangements are good.

## 7. Conclusions

In this paper, we presented a new attempt to lay ballastless track on long-span cable-stayed bridges, and the arrangements of the ballastless tracks on long-span cable-stayed bridges still need to be investigated further. Based on a long-span cable-stayed bridge, an equivalent segmental model was designed and built for this paper, and the ballastless tracks were laid on the segmental model with different arrangements. The full-scale fatigue tests and post-fatigue loading tests were carried out to research the mechanical behaviors and fatigue performances of ballastless tracks laid on long-span cable-stayed bridges with different arrangements. Some conclusions are as follows:

1. For ballastless tracks laid on long-span cable-stayed bridges, a rubber isolation layer can be used to improve mechanical behaviors and prevent the occurrence of the breakdowns such as voids and gaps at the interlayers.
2. For ballastless tracks with the rubber isolation layers, the stability and reliability of the ballastless tracks with different arrangements can be ensured under the standard fatigue loading.
3. With the increase of the post-fatigue static loading, the interlayer behaviors show apparent nonlinearity, which are determined by the structural characteristics of the rubber isolation layer.
4. The deformations and pressure variations at the interlayers are mutually corroborated so that the reliability of the test results can be ensured. Moreover, the structural behaviors and fatigue performances of ballastless tracks with different arrangements laid on long-span cable-stayed bridges are good.

## Figures and Tables

**Table 1:** Distance between the measuring sensors and the loading positions (m).

Number of the Cross-Section	Location of the Cross-Section	Loading Locations on the Ballastless Tracks	
		Ballastless Track Labeled as J1	Ballastless Track Labeled as J2
Cross-section #1	Near the slab end	3.29 m	1.28 m
Cross-section #2	1/4 of the slab	1.93 m	0.08 m
Cross-section #3	Center of the slab	0.45 m	1.56 m
Cross-section #4	3/4 of the slab	1.03 m	3.04 m
Cross-section #5	Near the slab end	2.39 m	4.40 m
